# Glucosamine as a Treatment for Osteoarthritis: What If It’s True?

**DOI:** 10.3389/fphar.2022.820971

**Published:** 2022-03-17

**Authors:** Thierry Conrozier, Thomas Lohse

**Affiliations:** Department of Rheumatology, Hôpital Nord Franche-Comté, Belfort, France

**Keywords:** glucosamine, OA, O-GlcNAcylation, treatment, low-grade inflammation, chondroprotective, cardio-vascular mortality, metabolic syndrom

## Abstract

No disease-modifying treatments are currently available for osteoarthritis (OA). While many therapeutic approaches are now being investigated it is ethical to resort to alternative solutions as that we already possess. There are many reasons for thinking that, at sufficiently high doses, glucosamine (GlcN) sulphate possesses a clinically relevant effect on OA pain. Wide inter-individual variations in the symptomatic effects of GlcN are explained by the extreme variability of its bioavailability. In studies evaluating its structure-modifying effect, GlcN was more effective than placebo in reducing the rate of joint space narrowing in patients with knee OA. More recent data suggest that GlcN may be effective in the primary prevention of OA in sportsmen. There is no controversy concerning the safety of GlcN which does not differ to that of placebo. Several studies have recently revealed an unexpected effect of GlcN on cardiovascular mortality. After adjusting for confounding factors, the regular consumption of GlcN correlated with a 27% reduction in mortality and a 58% reduction in deaths from cardiovascular causes. These data confirm animal studies demonstrating a protective effect of GlcN against cancer and cardiovascular diseases due to modulation of the O-GlcNAcylation pathway. Disorders in O-GlcNAcylation are involved in diabetes, obesity and cancers, which all feature chronic low-grade inflammation (CLGI). By regulating CLGI, GlcN may be beneficial to the symptoms of OA, its outcome and to that of the concomitant chronic pathologies, making GlcN as a valuable candidate for the treatment of OA in patients with metabolic syndrome, diabetes or cardiovascular diseases.

## Introduction

Osteoarthritis (OA) is a major cause of pain and disability in subjects older than 50 years with a significant impact on physical performance and quality of life. Its prevalence is increasing worldwide with the aging of the population and the increase in risk factors such as obesity, resulting in a significant public health problem and socioeconomic burden. However, OA is not the exclusive preserve of elderly and also affects younger people, especially those having risk factors such as overweight, metabolic syndrome or joint injury (Kloppenburg and Berenbaum, 2020). The pathophysiology of OA is complex and involves, at various levels, interactions between mechanical, genetic, metabolic and inflammatory mechanisms. Anatomically OA is characterized by articular cartilage breakdown, subchondral bone remodelling and synovial low-grade inflammation. To date no disease-modifying therapeutics are currently available for OA due to an insufficient understanding of the pathogenesis, and a delay in the therapeutic management, due to the lack of a sufficiently sensitive biomarker allowing to make the diagnosis during the early asymptomatic phase of the disease. At an advanced stage the treatment of OA is mainly based on symptomatic measures or joint replacement surgery. On the other hand, if the diagnosis is made early, developing treatments to slow the progression of joint degradation is a reasonable goal. Thanks to recent achievements in understanding the causes of the cartilage degradation many therapeutic approaches are now being investigated (Hochberg et al., 2019; Ghouri and Conaghan, 2019; Stevens et al., 2019; Thorup et al., 2020; Yazici et al., 2020; Fernández-Martín et al., 2021). However, we cannot hope for a structure-modifying treatment to be marketed for several years. In clinical daily practice all the care-givers must legitimately ask them the following question: Is it ethical not to bring forward patients anything at all (except symptomatic treatments, most of which having frequent and sometimes serious side effects) until a real chondroprotective agent has been launched on the market?

This mini review is not a systematic review and does not aim to provide all the data published on this topic, but, as the title indicates, is intended to ask a question. Is it possible that glucosamine actually has a beneficial effect on osteoarthritis?

The committed stance of the authors is to emphasize that, despite controversies, a number of data suggest that the long-term prescription of glucosamine could be beneficial for OA patients, both symptomatically and in terms of cardiovascular status.

Glucosamine (2-amino-2-deoxy-β-d-glucopyranose) is an endogenous amino-monosaccharide synthesized from glucose and utilized for biosynthesis of glycoproteins and glycosaminoglycans of joint cartilage which, when administered orally as the sulphate or hydrochloride, is considered to be a medicinal product of the class of symptomatic slow acting drugs for OA (SYSADOA). It has been used as an OA disease-modifying medicine for over 50 years (Vetter, 1965). Glucosamine is recommended for the treatment of knee by most European scholarly societies (Jordan et al., 2003; Bernetti et al., 2019; Bruyère et al., 2019; Sellam et al., 2020), but not by US ones (Bannuru et al., 2019; Kolasinski et al., 2020). However, with the exception of ESCEO, which recommend glucosamine as first-line treatment along with other SYSADOAs (Jordan et al., 2003), it is commonly considered as an adjunctive treatment, of modest symptomatic efficacy, mainly used for its excellent safety and because of its sparing effect on analgesic medications, notably NSAIDs, potentially much more dangerous (Dougados, 2006). However recent data, in particular studies demonstrating decreased cardiovascular mortality in patients receiving long-term treatment with glucosamine (Li et al., 2020) and the discovery of its mechanism of action on chronic inflammation (Herrero-Beaumont and Largo, 2020), have changed the perspective on this molecule, the indications of which could be considerably expanded.

## Symptomatic Effects in OA

Despite contradictory results in studies, there are numerous reasons for thinking that, at sufficiently high doses, some medicines containing glucosamine sulphate possess a significant and clinically relevant effect in OA (Gregori et al., 2018). Several randomised controlled studies have shown a symptomatic effect superior to placebo (Noack et al., 1994) and not inferior to that of an NSAID (Müller-Fassbender et al., 1994). The most recent meta-analyses confirm the symptomatic efficacy of pharmaceutical grade glucosamine sulphate, with results showing a standardised mean difference ranging from −0.29 [95% CI −0.49 to −0.09] (Sellam et al., 2020) and −0.35 [−0.54 to −0.16] (Knapik et al., 2018; Beaudart et al., 2020; Ton et al., 2020), which evidences a moderate effect not significantly different from results obtained with other classes of oral symptomatic OA drugs, notably the NSAIDs. OARSI classifies the efficacy of glucosamine as “uncertain,” due to a very variable effect size ranging from 0.17 [0.05–0.28] (low) to 0.47 [0.23–0.72] (moderate) depending on study quality and especially depending on the type of glucosamine studied (hydrochloride *versus* sulphate, pharmaceutical grade or not) (Bannuru et al., 2019). The key point, which seems to confirm the objective nature of the improvement, is that the analgesic effect occurs after several weeks’ treatment (Conrozier et al., 2019) and that it persists for several weeks after discontinuation of treatment, which does not resemble a placebo response but is rather the definition of an SYSADOA. One can nevertheless question the clinically relevant nature of the clinical improvement, which is often found to be lower than the MCII (Minimum Clinically Important Improvement). However, beyond the data from the studies, the fact that the commercial market for glucosamine is growing despite the wide publicity given to negative clinical trials, is consistent with the possibility that some patients obtain significant clinical benefits (McCarty et al., 2019), as in the PREDOA study where a clinically relevant improvement was noted in 63% of 2,030 patients treated for 6 months (Conrozier et al., 2019). One explanation of the wide interindividual variation in the symptomatic effects of glucosamine is the extreme variability of its bioavailability. (Asthana et al., 2020) Following oral administration, mean bioavailability is low, in the order of 25%, but with considerable variation between subjects according to a ratio of 1:100 (Li et al., 2020). The reasons for these variations remain unknown but an animal study has shown a 1.7-fold increase in plasma concentrations when glucosamine is taken on an empty stomach and in the evening, suggesting an influence of circadian rhythm (Seto et al., 2020). It is also important to know that the concomitant administration of glucosamine and chondroitin reduces the absorption of the former substance by 58% (Jackson et al., 2010). This effect of competition in cells of the intestinal is not without consequence because while glucosamine and chondroitin are both effective on the symptoms of OA, this is not the case when the two are combined, as highlighted by the meta-analysis of Zhu et al. (2018). Following absorption, synovial fluid concentrations of glucosamine are approximately double those in the serum. A possible hypothesis is that sufficient doses of oral glucosamine may have a positive effect on the synthesis of hyaluronic acid by the human synovial cells, as suggested by *in vitro* studies (Igarashi et al., 2011).

## Structural Effects in OA

The structural effect of oral glucosamine in knee OA was assessed in double-blind, randomized, controlled trials, lasting at least 1 year and reporting as outcome measures both the symptom severity and the radiological joint space width progression over time (Reginster et al., 2001; Pavelká et al., 2002; Poolsup et al., 2005). Glucosamine sulphate was found to be more effective than placebo in reducing the rate of joint space narrowing at the tibiofemoral compartment in patients with knee OA, slowing its progression by 54% (RR = 0.46; 95%CI 0.28–0.73; *p* = 0.001) with NNT (number of subjects needed to treat to reach the objective) of 9 (95% CI 6 – 20) (Pavelká et al., 2002). Although the figures appear very encouraging (in comparison the NNT of alendronate for prevention of vertebral fracture is 16 for secondary prevention and 50 for primary prevention) (Holder and Kerley, 2008), they may logically be considered as non-clinically relevant when limiting results to the rate of joint space narrowing compared with placebo, which is only 1/10th of a millimetre per year (−0.31 mm [95% CI −0.48 to −0.13]) for placebo and −0.06 mm [−0.22 to 0.09)] for glucosamine sulphate over a 3-year period) (Reginster et al., 2001). Nevertheless these values are not lower than those obtained by Sprifermin (recombinant FGF-18) (Hochberg et al., 2019) which is nonetheless often presented as the next generation “chrondroprotector”. It is particularly interesting to note that in patients with a high cartilage turnover, demonstrated by a very high level of urinary CTX-II before treatment, Christgau et al. noted a significant decrease in CTX-II levels after 12 months of glucosamine treatment and that the change in this level was predictive of radiologically-visible joint narrowing at 3 years (R = 0.43; *p* < 0.05) (Christgau et al., 2004). These data tend to support an effect, admittedly modest, but statistically significant, of glucosamine in secondary prevention of exacerbation of OA of the knee.

More recent data even suggest that glucosamine may be effective in the primary prevention of OA. This was the finding of a Japanese team who studied the effect of glucosamine on markers of the synthesis (CP II) and breakdown (u-CTX-II) of type II collagen in young athletes (rugby and soccer players) and in non-athletes paired for age (Nagaoka et al., 2019). Before treatment, levels of biomarkers were significantly higher in the athletes. After 3 months of treatment with glucosamine 1.5 or 3 g/day, the levels of biomarkers in the athletes had returned to the levels of the control subjects, and 3 months after discontinuation of treatment these had again increased. In a second study (Tsuruta et al., 2018) they randomised the athletes to receive either a dietary supplement containing 2 g of glucosamine or a placebo. After 3 months of treatment the u-CTX-II levels had significantly decreased in the glucosamine group but not in the placebo group. These 2 studies suggest that the administration of high doses of glucosamine (2–3 g per day) has a positive effect on cartilage metabolism *in vivo*, thus confirming the effect observed *in vitro*, where glucosamine inhibits matrix metalloproteinase production and chondrocyte apoptosis (Henrotin et al., 2012) and stimulates haeme oxygenase-1 (HO-1), a key enzyme regulating oxidative stress (Rousset et al., 2013).

## Safety

In contrast to its efficacy, there is no controversy concerning the safety in use of glucosamine; all meta-analyses and systematic reviews conclude that its safety is excellent, not different from placebo (Zhu et al., 2018). Conventionally, glucosamine is not recommend in patients with shellfish allergy due to its origin (the chitin of crustaceans) but reports of allergic phenomena, skin or respiratory, are not found to any significant extent in the literature. As glucosamine is a molecule similar to glucose its use in diabetics has been discussed, but studies showed no effect on blood glucose levels and hyperinsulinism at the doses used, whether in healthy subjects or diabetics (Simon et al., 2011).

## Effect on Mortality

Several studies (Simon et al., 2011; King and Xiang, 2020; Li et al., 2020) have recently revealed an unexpected effect of glucosamine. Cardiovascular mortality and mortality all causes, was studied from the clinical data of 16,686 subjects, participants in the American national Survey on health and nutrition from 1999 to 2010. Of these participants, 4% had been taking glucosamine or chondroitin for 1 year or more. During the study period, 20% of participants died. After adjusting for age, sex, race, education level, smoking and physical activity, the intake of glucosamine/chondroitin correlated to a 27% reduction in mortality all causes (HR = 0.73; [0.57 to 0.93]) and a 58% reduction in deaths from cardiovascular causes (HR = 0.42; [0.23 to 0.75]). A Chinese study in a cohort of 500,000 patients followed up for 9 years (Li et al., 2020) found similar results, although slightly less convincing. A notable finding was that the protective effect of glucosamine was particularly marked in smokers (*p* = 0.0008). These results in humans only confirm the very numerous animal studies which demonstrate a protective effect of glucosamine against cancer (lung and colorectal) and cardiovascular diseases (Largo et al., 2009). However, the doses used in animal studies are generally much higher than those authorised in humans.

It is thought that the beneficial effect of glucosamine is due to modulation of the O-GlcNAcylation pathway (Herrero-Beaumont and Largo, 2020) which is a reversible post-translational modification, analogous to phosphorylation, controlling the activity, location or stability of proteins, depending on glucose availability, by adding N-Acetyl-glucosamine to the serine or threonine residues of cytosolic or nuclear proteins. Tardio et al. showed that in human OA cartilage there is an accumulation of O-GlcNAcylated proteins associated to an alteration in the expression of the enzymes that regulate this glycosylation and an overexpression of hypertrophic differentiation markers in chondrocytes (Tardio et al., 2014). Changes in O-GlcNAcylation are involved in human disease such as diabetes, obesity and some cancers, pathologies which all feature chronic low-grade inflammation causing numerous complications. Hyperglycaemia combined with metabolic syndrome is known to enhance inflammatory processes via oxidative stress and the abnormally high levels of the O-GlcNAc protein which increase the transcriptional activity of nuclear factor kappa B (NFĸB), the cause of diabetic complications (Baudoin and Issad, 2015). However now we know that there is a close relationship which links OA, metabolic syndrome, diabetes, obesity and the resulting cardiovascular diseases. It is therefore tempting to think that by regulating the chronic low-grade inflammation which is common to all these pathologies, glucosamine may be beneficial to the symptoms of OA, its outcome and also to that of the concomitant chronic pathologies. As it also acts on oxidative stress by stimulating the production of HO-1 (Rousset et al., 2013), and that its safety is well known, glucosamine is today a valuable candidate for the treatment of OA in patients with metabolic syndrome, diabetes or a history of cardiovascular disease all of which contra-indicate the use of NSAIDs and intra-articular corticosteroids.

## Conclusion

Well-known and used as a SYSADOA for many years, glucosamine has been given a new lease of life with the demonstration of its beneficial effect in some chronic degenerative pathologies such as diabetes, obesity and atherosclerosis. While we know that its activity regulating O-GlcNAcylation is probably the reason for its effect on chronic inflammation, there are nevertheless numerous points requiring clarification such as optimal posology, the choice of salt (sulphate or hydrochloride), treatment duration, bioavailability and structural efficacy in primary or secondary prevention, before glucosamine can be considered as an anti-osteoarthritic drug of the future.

## References

[B1] Asthana C. Peterson G. M. Shastri M. D. Jones G. Patel R. P. (2020). Variation in Plasma Levels of Glucosamine with Chronic Dosing: A Possible Reason for Inconsistent Clinical Outcomes in Osteoarthritis. Clin. Ther. 42 (8), e140–e149. 10.1016/j.clinthera.2020.06.009 32713600

[B2] Bannuru R. R. Osani M. C. Vaysbrot E. E. Arden N. K. Bennell K. Bierma-Zeinstra S. M. A. (2019). OARSI Guidelines for the Non-surgical Management of Knee, Hip, and Polyarticular Osteoarthritis. Osteoarthr. Cartil. 27 (11), 1578–1589. 10.1016/j.joca.2019.06.011 31278997

[B3] Baudoin L. Issad T. (2015). O-GlcNAcylation and Inflammation: A Vast Territory to Explore. Front. Endocrinol. (Lausanne) 5, 235. 10.3389/fendo.2014.00235 25620956 PMC4288382

[B4] Beaudart C. Lengelé L. Leclercq V. Geerinck A. Sanchez-Rodriguez D. Bruyère O. (2020). Symptomatic Efficacy of Pharmacological Treatments for Knee Osteoarthritis: A Systematic Review and a Network Meta-Analysis with a 6-Month Time Horizon. Drugs 80 (18), 1947–1959. 10.1007/s40265-020-01423-8 33074440 PMC7716887

[B5] Bernetti A. Mangone M. Villani C. Alviti F. Valeo M. Grassi M. C. (2019). Appropriateness of Clinical Criteria for the Use of SYmptomatic Slow-Acting Drug for OsteoArthritis (SYSADOA). A Delphi Method Consensus Initiative Among Experts in Italy. Eur. J. Phys. Rehabil. Med. 55 (5), 658–664. 10.23736/S1973-9087.19.05633-8 31106560

[B6] Bruyère O. Honvo G. Veronese N. Arden N. K. Branco J. Curtis E. M. (2019). An Updated Algorithm Recommendation for the Management of Knee Osteoarthritis from the European Society for Clinical and Economic Aspects of Osteoporosis, Osteoarthritis and Musculoskeletal Diseases (ESCEO). Semin. Arthritis Rheum. 49 (3), 337–350. 10.1016/j.semarthrit.2019.04.008 31126594

[B7] Christgau S. Henrotin Y. Tankó L. B. Rovati L. C. Collette J. Bruyere O. (2004). Osteoarthritic Patients with High Cartilage Turnover Show Increased Responsiveness to the Cartilage Protecting Effects of Glucosamine Sulphate. Clin. Exp. Rheumatol. 22 (1), 36–42. 15005002

[B8] Conrozier T. Renevier J. L. Parisaux J. M. Balblanc J. C. (2019). Predictive Factors of Adherence to an Association of Glucosamine Sulfate, Copper, and Ginger Extracts in Patients with Symptomatic Osteoarthritis: a Prospective Open-Label French Noninterventional Study (The PREDOA Study). Patient Prefer Adherence 13 (13), 915–921. 10.2147/PPA.S200892 31239649 PMC6559715

[B9] Dougados M. (2006). Symptomatic Slow-Acting Drugs for Osteoarthritis: what Are the Facts? Jt. Bone Spine 73 (6), 606–609. 10.1016/j.jbspin.2006.09.008 17126058

[B10] Fernández-Martín S. López-Peña M. Muñoz F. Permuy M. González-Cantalapiedra A. (2021). Bisphosphonates as Disease-Modifying Drugs in Osteoarthritis Preclinical Studies: a Systematic Review from 2000 to 2020. Arthritis Res. Ther. 23 (1), 60. 10.1186/s13075-021-02446-6 33618776 PMC7898436

[B11] Ghouri A. Conaghan P. G. (2019). Update on Novel Pharmacological Therapies for Osteoarthritis. Ther. Adv. Musculoskelet. Dis. 11, 1759720X19864492. 10.1177/1759720X19864492 PMC6651659 31384314

[B12] Gregori D. Giacovelli G. Minto C. Barbetta B. Gualtieri F. Azzolina D. (2018). Association of Pharmacological Treatments with Long-Term Pain Control in Patients with Knee Osteoarthritis: A Systematic Review and Meta-Analysis. JAMA 320 (24), 2564–2579. 10.1001/jama.2018.19319 30575881 PMC6583519

[B13] Henrotin Y. Mobasheri A. Marty M. (2012). Is There Any Scientific Evidence for the Use of Glucosamine in the Management of Human Osteoarthritis? Arthritis Res. Ther. 14 (1), 201. 10.1186/ar3657 22293240 PMC3392795

[B14] Herrero-Beaumont G. Largo R. (2020). Glucosamine and O-GlcNAcylation: a Novel Immunometabolic Therapeutic Target for OA and Chronic, Low-Grade Systemic Inflammation? Ann. Rheum. Dis. 79 (10), 1261–1263. 10.1136/annrheumdis-2020-217454 32554393

[B15] Hochberg M. C. Guermazi A. Guehring H. Aydemir A. Wax S. Fleuranceau-Morel P. (2019). Effect of Intra-articular Sprifermin vs Placebo on Femorotibial Joint Cartilage Thickness in Patients with Osteoarthritis: The FORWARD Randomized Clinical Trial. JAMA 322 (14), 1360–1370. 10.1001/jama.2019.14735 31593273 PMC6784851

[B16] Holder K. K. Kerley S. S. (2008). Alendronate for Fracture Prevention in Postmenopause. Am. Fam. Phys. 78 (5), 579–581. 18788232

[B17] Igarashi M. Kaga I. Takamori Y. Sakamoto K. Miyazawa K. Nagaoka I. (2011). Effects of Glucosamine Derivatives and Uronic Acids on the Production of Glycosaminoglycans by Human Synovial Cells and Chondrocytes. Int. J. Mol. Med. 27 (6), 821–827. 10.3892/ijmm.2011.662 21455564

[B18] Jackson C. G. Plaas A. H. Sandy J. D. Hua C. Kim-Rolands S. Barnhill J. G. (2010). The Human Pharmacokinetics of Oral Ingestion of Glucosamine and Chondroitin Sulfate Taken Separately or in Combination. Osteoarthr. Cartil. 18 (3), 297–302. 10.1016/j.joca.2009.10.013 PMC2826597 19912983

[B19] Jordan K. M. Arden N. K. Doherty M. Bannwarth B. Bijlsma J. W. Dieppe P. (2003). EULAR Recommendations 2003: an Evidence Based Approach to the Management of Knee OA: Report of a Task Force of the Standing Committee for International Clinical Studies Including Therapeutic Trials (ESCISIT). Ann. Rheum. Dis. 62 (12), 1145–1155. 10.1136/ard.2003.011742 14644851 PMC1754382

[B20] King D. E. Xiang J. (2020). Glucosamine/Chondroitin and Mortality in a US NHANES Cohort. J. Am. Board Fam. Med. 33 (6), 842–847. 10.3122/jabfm.2020.06.200110 33219063 PMC8366581

[B21] Kloppenburg M. Berenbaum F. (2020). Osteoarthritis Year in Review 2019: Epidemiology and Therapy. Osteoarthr. Cartil. 28 (3), 242–248. 10.1016/j.joca.2020.01.002 31945457

[B22] Knapik J. J. Pope R. Hoedebecke S. S. Schram B. Orr R. Lieberman H. R. (2018). Effects of Oral Glucosamine Sulfate on Osteoarthritis-Related Pain and Joint-Space Changes: Systematic Review and Meta-Analysis. J. Spec. Oper. Med. 18 (4), 139–147. 10.55460/AUC0-QM7H 30566740

[B23] Kolasinski S. L. Neogi T. Hochberg M. C. Oatis C. Guyatt G. Block J. (2020). 2019 American College of Rheumatology/Arthritis Foundation Guideline for the Management of Osteoarthritis of the Hand, Hip, and Knee. Arthritis Care Res. (Hoboken) 72 (2), 149–162. 10.1002/acr.24131 31908149 PMC11488261

[B24] Largo R. Martínez-Calatrava M. J. Sánchez-Pernaute O. Marcos M. E. Moreno-Rubio J. Aparicio C. (2009). Effect of a High Dose of Glucosamine on Systemic and Tissue Inflammation in an Experimental Model of Atherosclerosis Aggravated by Chronic Arthritis. Am. J. Physiol. Heart Circ. Physiol. 297 (1), 268–276. 10.1152/ajpheart.00142.2009 19411287

[B25] Li Z. H. Gao X. Chung V. C. Zhong W. F. Fu Q. Lv Y. B. (2020). Associations of Regular Glucosamine Use with All-Cause and Cause-specific Mortality: a Large Prospective Cohort Study. Ann. Rheum. Dis. 79 (6), 829–836. 10.1136/annrheumdis-2020-217176 32253185 PMC7286049

[B26] McCarty M. F. O'Keefe J. H. DiNicolantonio J. J. (2019). Glucosamine for the Treatment of Osteoarthritis: The Time Has Come for Higher-Dose Trials. J. Diet. Suppl. 16 (2), 179–192. 10.1080/19390211.2018.1448920 29667462

[B27] Müller-Fassbender H. Bach G. L. Haase W. Rovati L. C. Setnikar I. (1994). Glucosamine Sulfate Compared to Ibuprofen in Osteoarthritis of the Knee. Osteoarthr. Cartil. 2 (1), 61–69. 10.1016/s1063-4584(05)80007-x 11548225

[B28] Nagaoka I. Tsuruta A. Yoshimura M. (2019). Chondroprotective Action of Glucosamine, a Chitosan Monomer, on the Joint Health of Athletes. Int. J. Biol. Macromol. 132, 795–800. 10.1016/j.ijbiomac.2019.03.234 30940583

[B29] Noack W. Fischer M. Förster K. K. Rovati L. C. Setnikar I. (1994). Glucosamine Sulfate in Osteoarthritis of the Knee. Osteoarthr. Cartil. 2 (1), 51–59. 10.1016/s1063-4584(05)80006-8 11548224

[B30] Pavelká K. Gatterová J. Olejarová M. Machacek S. Giacovelli G. Rovati L. C. (2002). Glucosamine Sulfate Use and Delay of Progression of Knee Osteoarthritis: a 3-year, Randomized, Placebo-Controlled, Double-Blind Study. Arch. Intern. Med. 162 (18), 2113–2123. 10.1001/archinte.162.18.2113 12374520

[B31] Poolsup N. Suthisisang C. Channark P. Kittikulsuth W. (2005). Glucosamine Long-Term Treatment and the Progression of Knee Osteoarthritis: Systematic Review of Randomized Controlled Trials. Ann. Pharmacother. 39 (6), 1080–1087. 10.1345/aph.1E576 15855241

[B32] Reginster J. Y. Deroisy R. Rovati L. C. Lee R. L. Lejeune E. Bruyere O. (2001). Long-term Effects of Glucosamine Sulphate on Osteoarthritis Progression: a Randomised, Placebo-Controlled Clinical Trial. Lancet 357 (9252), 251–256. 10.1016/S0140-6736(00)03610-2 11214126

[B33] Rousset F. Nguyen M. V. Grange L. Morel F. Lardy B. (2013). Heme Oxygenase-1 Regulates Matrix Metalloproteinase MMP-1 Secretion and Chondrocyte Cell Death via Nox4 NADPH Oxidase Activity in Chondrocytes. PLoS One 8 (6), e66478. 10.1371/journal.pone.0066478 23840483 PMC3688771

[B34] Sellam J. Courties A. Eymard F. Ferrero S. Latourte A. Ornetti P. (2020). Recommendations of the French Society of Rheumatology on Pharmacological Treatment of Knee Osteoarthritis. Jt. Bone Spine 87 (6), 548–555. 10.1016/j.jbspin.2020.09.004 32931933

[B35] Seto Y. Yoshihashi T. Tomonari M. To H. (2020). Absorption of Glucosamine Is Improved by Considering Circadian Rhythm and Feeding in Rats. Chronobiol. Int. 37, 1528. 10.1080/07420528.2020.1784189 32576047

[B36] Simon R. R. Marks V. Leeds A. R. Anderson J. W. (2011). A Comprehensive Review of Oral Glucosamine Use and Effects on Glucose Metabolism in normal and Diabetic Individuals. Diabetes Metab. Res. Rev. 27 (1), 14–27. 10.1002/dmrr.1150 21218504 PMC3042150

[B37] Stevens R. M. Ervin J. Nezzer J. Nieves Y. Guedes K. Burges R. (2019). Randomized, Double-Blind, Placebo-Controlled Trial of Intraarticular Trans-capsaicin for Pain Associated with Osteoarthritis of the Knee. Arthritis Rheumatol. 71 (9), 1524–1533. 10.1002/art.40894 30888737 PMC6772016

[B38] Tardio L. Andrés-Bergós J. Zachara N. E. Larrañaga-Vera A. Rodriguez-Villar C. Herrero-Beaumont G. (2014). O-linked N-Acetylglucosamine (O-GlcNAc) Protein Modification Is Increased in the Cartilage of Patients with Knee Osteoarthritis. Osteoarthr. Cartil. 22 (2), 259–263. 10.1016/j.joca.2013.12.001 24333294

[B39] Thorup A. S. Strachan D. Caxaria S. Poulet B. Thomas B. L. Eldridge S. E. (2020). ROR2 Blockade as a Therapy for Osteoarthritis. Sci. Transl. Med. 12, eaax3063. 10.1126/scitranslmed.aax3063 32938794

[B40] Ton J. Perry D. Thomas B. Allan G. M. Lindblad A. J. McCormack J. (2020). PEER Umbrella Systematic Review of Systematic Reviews: Management of Osteoarthritis in Primary Care. Can. Fam. Physi. 66 (3), e89–e98. PMC8302337 32165479

[B41] Tsuruta A. Horiike T. Yoshimura M. Nagaoka I. (2018). Evaluation of the Effect of the Administration of a Glucosamine-Containing Supplement on Biomarkers for Cartilage Metabolism in Soccer Players: A Randomized Double-Blind Placebo Controlled Study. Mol. Med. Rep. 18 (4), 3941–3948. 10.3892/mmr.2018.9396 30132529

[B42] Vetter G. (1965). Glucosamine in the Therapy of Degenerative Rheumatism. Dtsch Med. J. 516 (13), 446–449. 5319412

[B43] Yazici Y. McAlindon T. E. Gibofsky A. Lane N. E. Clauw D. Jones M. (2020). Lorecivivint, a Novel Intraarticular CDC-like Kinase 2 and Dual-Specificity Tyrosine Phosphorylation-Regulated Kinase 1A Inhibitor and Wnt Pathway Modulator for the Treatment of Knee Osteoarthritis: A Phase II Randomized Trial. Arthritis Rheumatol. 72 (10), 1694–1706. 10.1002/art.41315 32432388 PMC7589351

[B44] Zhu X. Sang L. Wu D. Rong J. Jiang L. (2018). Effectiveness and Safety of Glucosamine and Chondroitin for the Treatment of Osteoarthritis: a Meta-Analysis of Randomized Controlled Trials. J. Orthop. Surg. Res. 13 (1), 170. 10.1186/s13018-018-0871-5 29980200 PMC6035477

